# Supplementary material for: The Impact of Valvuloarterial Impedance on Left Ventricular Geometrical Change after Transcatheter Aortic Valve Replacement: A Comparison between Valvuloarterial Impedance and Mean Pressure Gradient
Source: J Clin Med. 2020 Sep 29;9(10):3143. doi: 10.3390/jcm9103143 (PMC7600915; doi:10.3390/jcm9103143)
Supplement: Supplementary file 1 [file jcm-09-03143-s001.pdf]

The Impact of Valvuloarterial Impedance on Left Ventricular Geometrical Change after Transcatheter Aortic Valve Replacement: A Comparison between Valvuloarterial Impedance and Mean Pressure Gradient

Satoshi Yamaguchi, Yuk Otaki, Balaji K. Tamarappoo, Tetsuya Ohira, Hiroki Ikenaga, Jun Yoshida, Tarun Chakravarty, John Friedman, Daniel Berman, Florian Rader, Robert J. Siegel, Raj Makkar and Takahiro Shiota

Table S1. Valve system of transthoracic aortic valve replacement.

Valve system	*N* = 301
SAPIEN™	51 (17)
SAPIEN XT™	102 (34)
SAPIEN 3™	95 (32)
Core valve™	27 (9)
Others	26 (8.6)

The deployed valve systems are displayed as number (%).
